# Effects of γ-polyglutamic acid on grassland sandy soil properties and plant functional traits exposed to drought stress

**DOI:** 10.1038/s41598-024-54459-1

**Published:** 2024-02-14

**Authors:** Tomasz Skalski, Ewelina Zając, Elżbieta Jędrszczyk, Katarzyna Papaj, Joanna Kohyt, Artur Góra, Anna Kasprzycka, Divine Shytum, Barbara Skowera, Agnieszka Ziernicka-Wojtaszek

**Affiliations:** 1 Tunneling Group, Biotechnology Center, Silesian University of Technology, Gliwice, Poland; 2 Department of Chemistry, Silesian University of Technology, Gliwice, Poland; 3 Department of Land Reclamation and Development, University of Agriculture, Krakow, Poland; 4 Department of Horticulture, University of Agriculture, Krakow, Poland; 5 Institute of Biology, Biotechnology and Environmental Protection, University of Silesia, Katowice, Poland; 6 Department of Ecology, Climatology and Air Protection, University of Agriculture, Krakow, Poland

**Keywords:** Climate sciences, Ecology, Environmental sciences, Natural hazards

## Abstract

The current study provides field experimental data that support the use of γ-polyglutamic acid (γ-PGA) in drought stress and proposes its application in grassland management. We hypothesized that water treatment combined with PGA application to sandy soil would reduce drought stress in grasslands more effectively than watering alone. A randomized block design was used, with three replicate watering blocks (no watering, weekly watering, and monthly watering) and PGA treatments at four different concentrations (0%, 0.3%, 1%, and 2% PGA). The results showed that PGA acts as a biostimulant, alleviating the effects of stress in plants by: (1) increasing the availability of ions, especially K^+^, Zn^2+^, Mn^2+^, Fe^2+/3+^, Ca^2+^, and Mg^2+^, as well as N-NH_4_^+^, and N-NO_3_^−^, (2) elongating plant roots, (3) increasing the aboveground biomass, (4) improving the resprouting capacity of the dominant grass *Nardus stricta*, and (5) improving the regeneration of dicotyledons. In the case of meadows on sandy soils, the use of low PGA concentrations (0.3% or 1%) was the most beneficial for the availability of macro- and microelements and improving the functional traits of plants. Irrigation had a greater effect than using PGA only for the dicotyledon to monocotyledon ratio.

## Introduction

A changing climate has the potential to alter the composition and structure of plant and soil communities and the interactions between them, so-called plant-soil feedbacks (PSFs)^1^. However, very little is known about the basic mechanisms and the consequences for PSF of climatic events such as drought. In particular, most studies have examined the role of soil microbial communities in PSF, focusing on examining the effects of those microbes involved in positively or negatively influencing plant performance^2^. Climate change creates shifts in microbial communities from bacterial on more humid soils to fungal on most dry soils and thus affects plant inputs into the soil subsystem via litter and rhizodeposits. As a consequence, the litter quality changes from high to low^3^, while the number of metabolic products that play an important role in the PSF relationship is also diminished.

It is known that bacteria and their metabolites play key roles in soil functioning and interact with plants and trophic chains^2^. One of the key metabolites is γ-polyglutamic acid (PGA), which is produced by *Lactobacillus* strains and is concentrated in the extracellular component of biofilms. Biofilms are complex microbial communities that are surrounded in an extracellular matrix containing many mucopolysaccharides, peptides, and anionic polyamides, whose function is related to the protection of *Lactobacillus* strains from the negative impacts of other bacteria, the maintenance of homeostasis, and the protection from external environmental conditions, such as drought stress, temperature changes, salinity, or UV radiation. Plants growing in the proximity of these biofilms also benefit and can even become bacterially dependent^4^. In agrocenoses, where many natural processes are broken^5^, crop plants are especially exposed to deficiencies in biofilm components. Therefore, in order to overcome this problem, modern agrostrategies include the treatment of plants with bacterial strains^6^ or fermentation products such as humic acids. These applications, however, are ineffective in the long term because of unfavorable agricultural soil conditions being magnified by chemical applications. One of the solutions to this problem is to produce bacterial metabolites in high amounts by biofermentation processes and apply them in the field^7^. A promising example of such a strategy is the biotechnological production of PGA in very high concentrations due to high bioreactor intensity and the acceleration of metabolic processes^8,9^.

PGA is a natural biofilm component, an optically active anionic polymer, which exists in two isoforms: α-PGA and γ-PGA. This polymer is built with repeating units of d- or l-glutamate connected via amide linkages that are formed between the α-amino and α/γ-carboxylic groups. Microorganisms produce three types of poly-glutamic acid: d-PGA, l-PGA (which are respectively composed of d-glutamate or l-glutamate units), and dl-PGA (in which d- and l-glutamate units are randomly connected^10^). PGA is characterized by its molecular weight, the carboxyl-group (α or γ) engaged in the amide bond, and by the ratio of d- to l-glutamic acid units^8^.

γ-PGA can protect microorganisms against phagocytic attacks and high salt concentrations, while also providing extracellular nutrient storage and promoting biofilm formation^8^. γ-PGA is water soluble, biodegradable, biocompatible, edible, and non-toxic to humans and the environment. It has the ability to bind metals and absorb water, and its biotechnological production for agricultural applications is becoming increasingly profitable. This polymer has many potential applications in the fields of pharmaceuticals, water and soil treatment, food, cosmetics, and agriculture^11–13^.

One of the most important applications of PGA is in agriculture. Due to the fact that γ-PGA and especially superabsorbent materials synthesized from PGA^14^ are able to hold a large amount of water, they can be used on crop fields as soil conditioners. Many studies have reported that γ-PGA plays an important role in plant growth and its regulation; for example, it raises the dry weight of plants such as cucumber, Chinese cabbage, spinach, and tomato^15–17^. γ-PGA can also be used to bind toxic metal ions and prevent plants from sequestering them. Due to its capacity to bind ions, γ-PGA can be used as a fertilizer, which delivers to plant rhizospheres cations^18^ such as Ca^2+^, Fe^2+^, Fe^3+^, Zn^2+^_,_ and Mn^2+^.

PGA can also be used to reduce salt-induced inhibition of plant growth. Under salt stress, the accumulation of toxic levels of cellular Na^+^ and restricted absorption of K^+^ were observed in plant cells. Interestingly, the foliar application of γ-PGA was shown to reduce ion-specific toxicity by decreasing the cellular accumulation of Na^+^, while increased K^+^ accumulation was observed^17^. In addition, recent studies showed that this polymer has beneficial effects on the availability of soil nitrogen, phosphorus, and potassium, as well as plant C and N metabolism^13^. Data from a limited number of single model plant species confirmed the acceleration of plant growth and increase in crop yield following PGA application. Moreover, γ-PGA stimulated plant N uptake and improved N soil availability by enhancing microbial and urease activity^15,16^. It is also known that γ-PGA can increase root biomass^15,19^ and that urease activity depends on PGA metabolites, such as glutamic acid or small homopeptides^13^. A recent experiment on the effect of PGA acceleration on the drought tolerance of *Brassica napus* seedlings confirmed that γ-PGA may induce a tendency towards plant tolerance to drought stress by promoting abscisic acid accumulation^20^.

In Europe, sandy soils are present mainly in Nordic countries and Baltic states, which reflects the glacial history of the continent^21^. In Poland, more than 60% of mineral soils have developed from sandy parent material. Sandy soils naturally show low water retention capacity and are prone to drought^22^. Semi-natural grasslands on light soils that are managed as meadows and pastures are especially endangered due to climate change^23,24^, which could result in reduced pastoral farming, crop loss^25^, and biodiversity loss^26^. *Nardus stricta* grasslands play an important role in supporting the diversity of the agricultural landscape^27^ and vulnerable plant preservation^28^. The consequences of drought in *N. stricta* grasslands can be observed in adjacent fields, meadows, and forest (broken ecosystem connectivity and metapopulation changes of pollinators^25^ and predators^29^ in agrocenoses). Therefore, intervention strategies aimed at mitigating the consequence of drought events in such ecosystems should be developed urgently.

The current study provides field experimental data which supports the use of γ-PGA in drought stress and proposes its application in grassland management. We hypothesize that water treatment combined with PGA application to light and sandy soil will reduce drought stress in grasslands more effectively than watering alone, by one of following mechanisms:

1. Increase in soil macro- and microelements’ availability to grasses and herbaceous plants,
2. Influence on plant root system development and aboveground biomass production,
3. Influence on the resprouting capacity of grasses (*Nardus stricta*) undergoing drought stress,
4. Supporting the regeneration of dicotyledons in grasslands.

## Materials and methods

### Study site

The survey was conducted in the vicinity of Staszów, Świętokrzyskie Voivodeship, Poland (50°35′N 21°11′E, 190 m a.s.l). The experimental site was a typical post-pasture grassland, mown irregularly only to sustain open areas. The site was surrounded by a mosaic of small arable areas, where mainly potatoes and cereals are cultivated. The grassland was classified in the *Nardo-Callunetea* class, with *Nardus stricta* as the dominant species, with *Potentilla erecta*, *Polygala vulgaris, Hieracium pilosella*, *Luzula campestris*, and *Rumex acetosella* co-domination. Thanks to the owners, the 3600 m^2^ experimental site was fenced to avoid any animal activity. The climate of the given region represents the transitional temperate zone, classified as Dfb (continental with warm summer) by the Köppen-Geiger system^30^ (adjacent to the north-east boundary of a moderately warm climate with uniform moisture content, Cfb). The average annual temperature in Staszów is 8.0 °C (− 3.0 °C in January and 18.3 °C in July). The multiyear average annual rainfall in this area is 570 mm.

**Figure 1:**
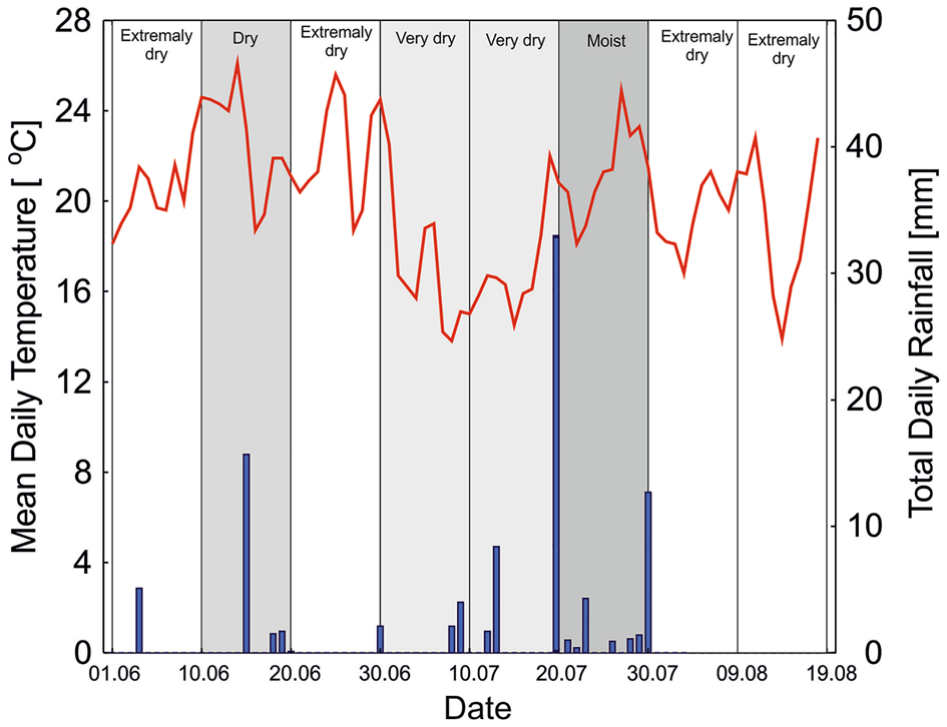
Mean daily temperature (orange line) and total daily rainfall (mm) during the period 1 June–19 August 2019 in 10-day sequences classified according to Selyaninov's Hydrothermal Coefficient (IMGW: https://danepubliczne.imgw.pl/data/dane_pomiarowo_obserwacyjne/: access August 2020).

Field experiments were conducted during the drought event of June–August 2019. A coincidental occurrence of drought and heavy precipitation was observed during the spring and summer in the investigated region (Fig. 1). An assessment of the occurrence of meteorological drought conditions in consecutive 10-day periods before and during the experiment was made on the basis of Selyaninov's Hydrothermal Coefficient, HTC^1^. During the experiment, four 10-day periods were classified as extremely dry (HTC = 0.0–0.25), three periods as very dry or dry (HTC = 0.46–0.82), and only one period was moist (HTC = 2.38), due to days with heavy rainfall.

### Soil parameters

The soil in the study area was loamy sand containing 81% sand, 16% silt, and 3% clay. The soil was highly acidic and generally nutrient poor (Table 1); especially low concentrations of Ca^2+^, Mg^2+^, K^+^, Zn^2+^, Mn^2+^, Fe^2+/3+^ were observed. However, the content of available P and N_min_ (N-NO_3_^−^ + N-NH_4_^+^) may indicate the application of fertilizers in the past.

**Table 1:** Mean, median, and range of the soil parameters in the reference experimental plots.

Parameter	Unit	Mean	Median	Min	Max	SD
pH	–	5.70	5.54	5.52	6.21	0.26
EC	µS cm^−1^	50.69	48.50	46.16	57.18	4.17
Ca^2+^	mg kg^−1^ soil	221.23	210.06	172.44	294.94	54.39
Mg^2+^	mg kg^−1^ soil	15.81	14.63	14.06	21.06	2.57
K^+^	mg kg^−1^ soil	35.09	36.50	27.38	39.63	5.25
Available P	mg kg^−1^ soil	57.63	14.16	9.48	302.82	101.38
N-NH_4_	mg kg^−1^ soil	8.75	10.94	3.28	10.94	3.46
N-NO_3_	mg kg^−1^ soil	6.84	8.75	2.19	8.75	2.97
Zn^2+^	mg kg^−1^ soil	1.66	1.77	1.21	1.78	0.22
Mn^2+^	mg kg^−1^ soil	2.34	2.94	0.47	3.12	1.16
Fe^2+/3+^	mg kg^−1^ soil	0.59	0.52	0.14	1.38	0.32

### Study design

**Figure 2:**
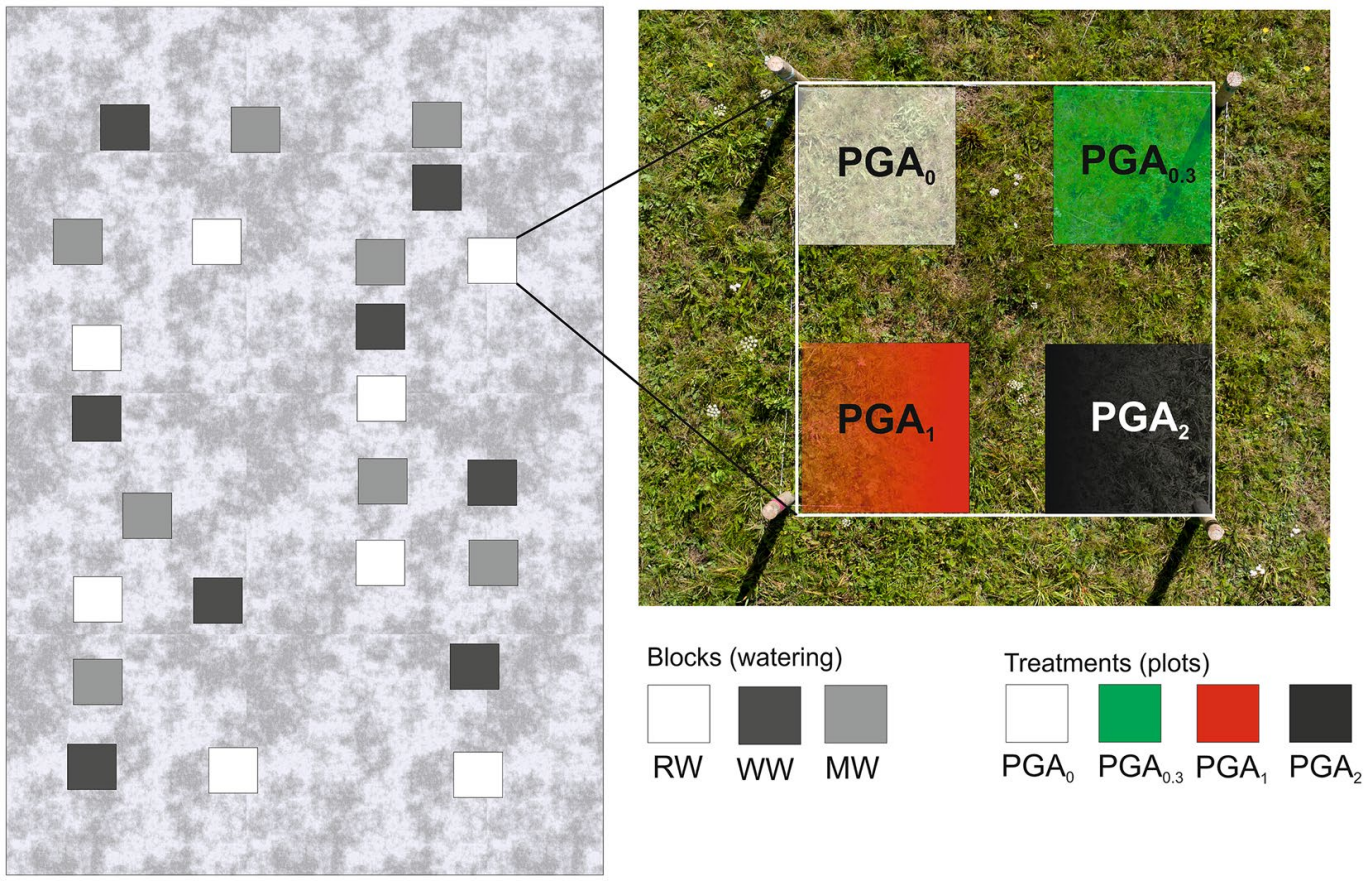
Study site design and spatial arrangement of 24 watering blocks and four PGA treatments in each block. Description of abbreviations in the text.

The experiment was carried out between 1 June and 15 August 2019. A randomized block design was used in the experiment, with three watering blocks replicated eight times and four PGA treatments at various concentrations. Eight blocks of 2.25 m^2^ (1.5 m × 1.5 m) were allocated to one of three different watering regimes (24 blocks in total). The spatial distance between blocks was greater than 10 m, while the spatial distribution was random. In each watering block, four plots of 0.25 m^2^ (0.5 m × 0.5 m) were allocated to different PGA treatments (96 plots in total) (Fig. 2). The mode of watering in each PGA treatment plot was the same.

### Blocks

The amount of water applied to each watering block was calculated on the basis of the water deficit from the multiyear (1991–2018) average monthly precipitation for the April–June period. The calculated water deficit (the difference between the average monthly precipitation and the April–June precipitation) since the beginning of the vegetation season was estimated to be approximately 42 L per square meter. A certain percentage of the water deficit was covered for each block: (1) RW (reference watering): no watering applied to establish very dry conditions, (2) WW (weekly watering): coverage of total (100%) calculated water deficit—2 L of water per treatment plot of 0.25 m^2^—applied weekly over a 6-week time period, 3) MW (monthly watering): coverage of 30% of calculated water deficit—2 L of water per treatment plot of 0.25 m^2^, applied twice at the beginning of experiment and after 4 weeks.

### Treatments

In each watering block, four PGA treatments were employed: (1) PGA_0_: reference, without PGA application, (2) PGA_2_: PGA concentration 2% (40 mL of PGA per m^2^), (3) PGA_1_: PGA concentration 1% (20 mL of PGA per m^2^), and (4) PGA_0.3_: PGA concentration 0.33% (6.6 mL of PGA per m^2^). Commercially available Ambiogel^®^ PGA (Ambioteco, Sztombergi, Poland), which contained 5% pure PGA, was applied once on the first day of the experiment.

### Plant functional traits

Plant species from abandoned fields were identified to species level by Prof. Elżbieta Jędrszczyk (Department of Horticulture, University of Agriculture, Krakow, Poland). All collected specimens were not under of law restriction or any permission. Voucher specimens were photographed and stored in the authors collection. On the last day of the experiment, plant samples (roots and shoots) were collected from each treatment plot from eight randomly distributed area units using the ring method. Eight random measurements (ring throws) for each plot using a ring of a given size (mentioned below), which was adopted as the minimum area of the estimated shoot variation, were carried out (768 samples in total). The plant's roots and stems were cut from 12.56 cm^2^ (measured with a loose-leaf ring, 4 cm in diameter) and rinsed gently with distilled water to remove soil remnants. The root max length was determined to be the maximum length from the root base. The aboveground dry biomass of the dominant grass species, *N. stricta*, was determined using the oven-dried biomass method^31^. The shoots were soaked in distilled water for 4 h and then dried at 80 °C for 24 h. In addition, the maximum aboveground plant height (MH) per area unit (4 cm diameter = 12.56 cm^2^) was measured as the maximum point reached by the highest plant.

Based on the measured plant parameters, two indices were calculated: (1) DFR—the ratio of dry to fresh shoots of *N. stricta*. A ring of 1 cm diameter = 0.78 cm^2^ was randomly thrown eight times, and the fresh and dry stems were visually calculated, (2) DMR—the ratio of dicotyledons to monocotyledons. The DMR was determined from the number of individual specimens per 78.5 cm^2^ ring size (10 cm diameter) to cover variations in the plant abundance. To obtain a random effect, the ring was thrown eight times for each treatment plot.

### Soil analysis

Soil samples for laboratory analysis were taken from the 0–10 cm layer of each treatment plot as pooled samples taken from triplicate samples (96 samples in all). The soil particle size distribution was determined by the hydrometer method according to the PN-R-04032 standard (Polish Committee for Standardization: Warszawa), while soil pH and electrical conductivity (EC) were measured using the potentiometric method with a soil/distilled water ratio of 1:5 (v/v). The levels of ammonium (N-NH_4_^+^) and nitrate (N-NO_3_^−^) nitrogen were determined by flow injection colorimetry analysis (FIAstar 5000, Foss), the levels of available phosphorus (P) and potassium (K) by the Egner-Riehm method, the available magnesium (Mg) by the Schachtschabel method. Phosphorus content in the solution was determined colorimetrically using UV–Vis spectrophotometer (Helios Beta UVB1002 E, Thermo Electron Corporation, Paisley, UK), while potassium and magnesium content by atomic absorption spectrometry (Varian SpectrAA-20). The level of calcium (Ca) and available microelements (Fe, Mn, and Zn) was determined by atomic absorption spectrometry after extraction into 0.03 M CH_3_COOH.

### Statistical analyses

The influence of watering and PGA treatments on the soil and plant parameters were compared using two-way ANOVA. The normality of the data was tested using the Shapiro–Wilk test. Post-hoc Duncan’s comparisons of the soil parameters were performed with a Bonferroni correction. All statistical analysis were performed using Statistica v.13 (TIBCO Software Inc., Palo Alto, CA, USA). Multivariate redundancy analysis (RDA) was carried out using non-standardized plant parameters. For soil parameters, PGA concentration, and watering (as a dummy variable), forward selection was employed to indicate the soil and treatment variables significantly describing the plant parameters’ variation. Multivariate statistical analyses were carried out using CANOCO software version 4.56. Values were considered to be statistically significant at p ≤ 0.05.

## Results

Two-way analysis of variance indicated that watering, either as a single parameter or in combination with PGA treatment, had no significant effect on soil parameters (Supplement 1). Only the PGA, as a single parameter, significantly influenced the soil macro- and microelement content and soil pH.

**Figure 3:**
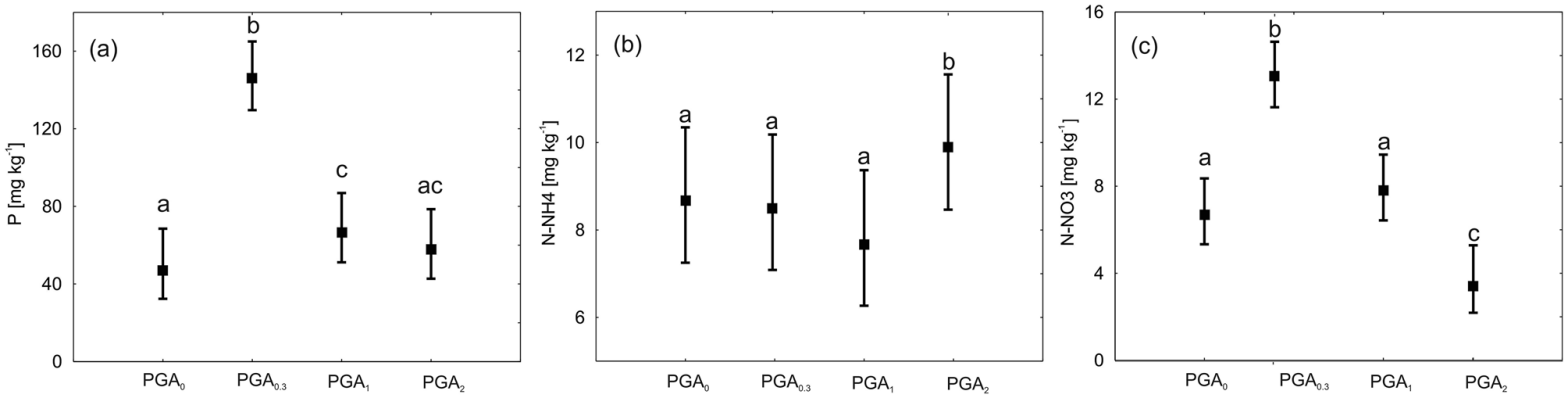
Mean comparison of the different concentrations of (**a**) available phosphorus (P), (**b**) ammonium nitrogen (N-NH4^+^) and (**c**) nitrate nitrogen (N-NO_3_^−^) compounds with respect to PGA treatments by Duncan tests (means with the same letter did not show significant differences).

The available phosphorus (P) and mineral nitrogen (N-NH_4_^+^ + N-NO_3_^−^) content were significantly enhanced by the presence of PGA with the results varying at different concentrations (Fig. 3). The available P content (Fig. 3a) in the soil was the highest when the lowest concentration of PGA was applied (PGA_0.3_). A similar pattern was observed for N-NO_3_^−^ (Fig. 3c). However, the highest N-NH_4_^+^ content was associated with the highest PGA concentration (PGA_0.3_) (Fig. 3b). Surprisingly, the reference (PGA_0_) content was always approximately at the level of the medium PGA concentration (PGA_1_).

**Figure 4:**
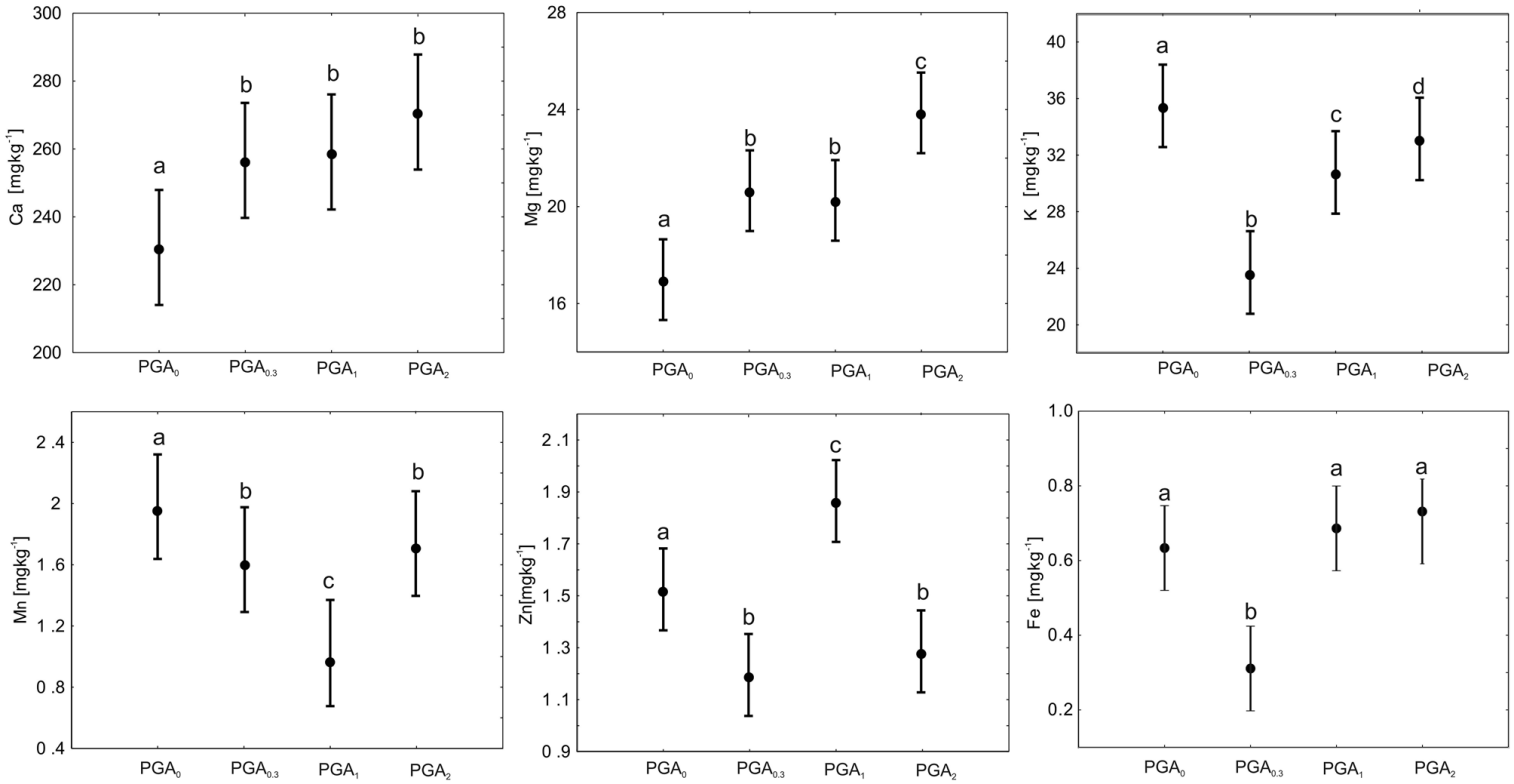
Mean comparison of the different concentrations of metal compounds with respect to PGA treatments by Duncan tests (means with the same letter did not show significant differences).

The variation in macro- and microelements in the soil is presented in Fig. 4. The content of the two divalent base cations in the soil, i.e., Ca^2+^ and Mg^2+^, significantly increased when the PGA was added (Fig. 4). The opposite effect was obtained for K^+^ and Mn^2+^, their distribution in the soil being decreased when PGA was applied. The Zn^2+^ concentration in the soil was reduced by PGA_2_ and PGA_0.3_ relative to PGA_0_, while the Fe^2+/3+^ content was reduced only at the lowest concentration of PGA (PGA_0.3_).

**Figure 5:**
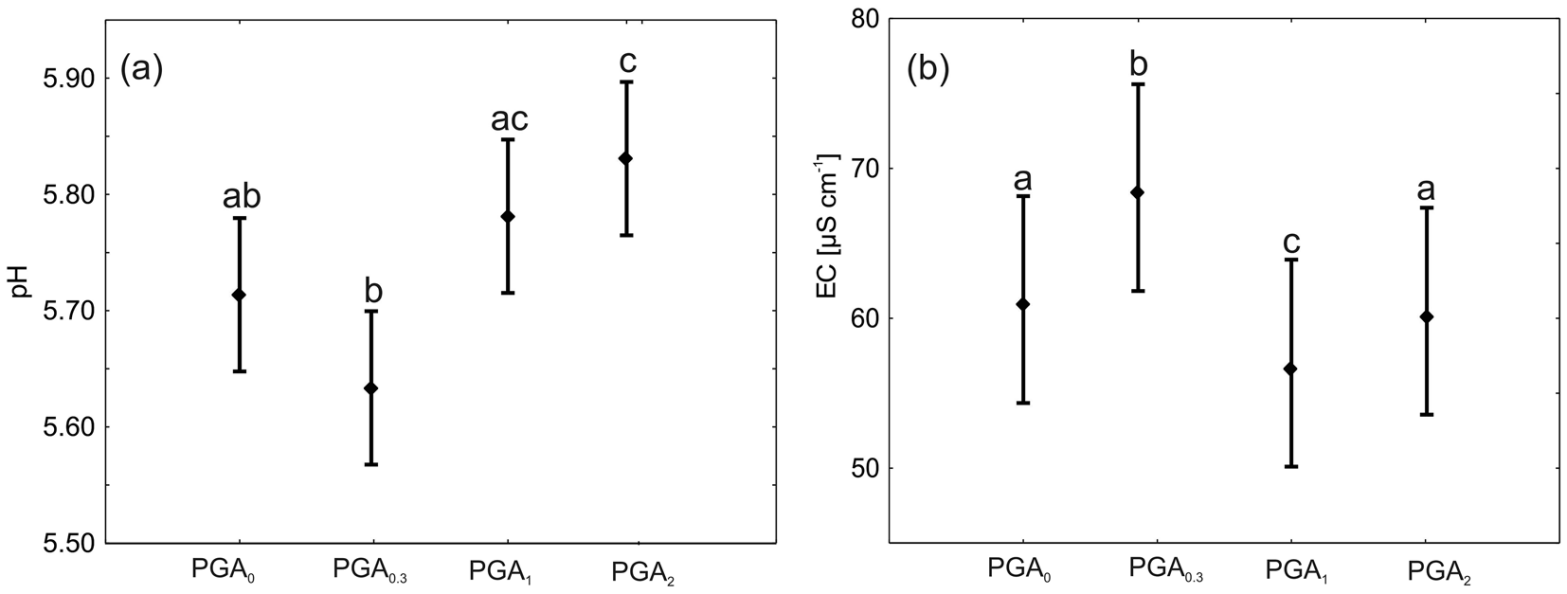
Mean soil pH (calculated on the basis of hydrogen ion concentration in mmol dm^−3^) (**a**) and electrical conductivity (EC) (**b**) with multiple comparison Duncan test (means with the same letter did not show significant differences).

High concentrations of PGA enhanced the soil pH values, while the lowest PGA concentration was responsible for the lowest pH values (Fig. 5a). Electrical conductivity (Fig. 5b), which reflected the concentration of soluble salts, was also related to the PGA content. The EC increased significantly when the PGA content was lowest.

Two-way ANOVA indicated that both factors in combination, i.e., watering and PGA concentration, significantly influenced plant functional traits under drought stress (Supplement 2).

**Figure 6:**
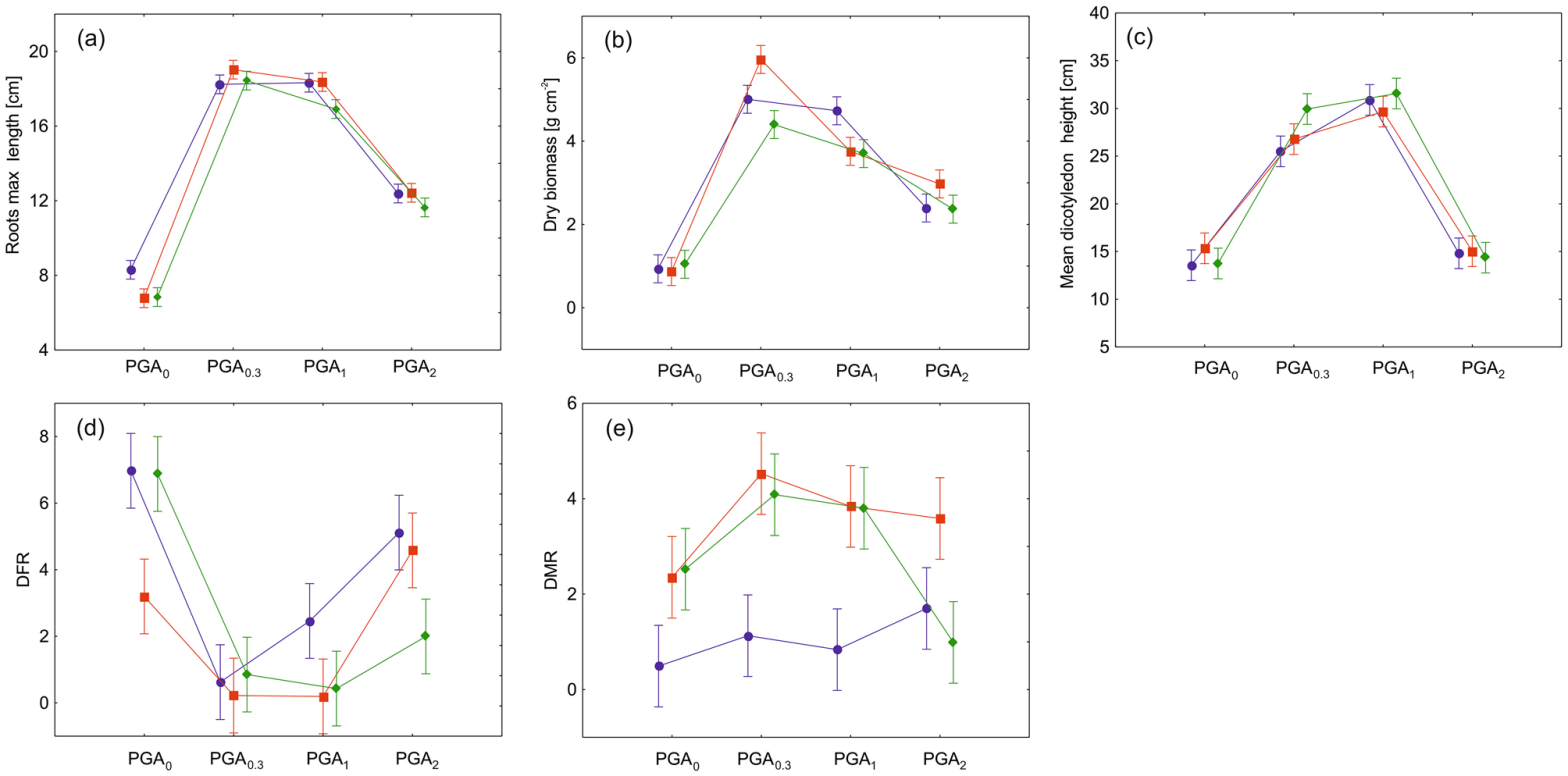
Mean and 95% of confidence interval values of grassland life traits: (**a**) *Nardus stricta* root length and (**b**) aboveground biomass; (**c**) maximum shoot height of dicotyledons; (**d**) DFR index; (**e**) DMR index per sample unit. Blue lines—reference watering (RW), green lines—monthly watering (MW), red lines—weekly watering (WW).

PGA applied at low or medium levels (PGA_0.3_, PGA_1_) significantly increased the mean root length (Fig. 6a) and aboveground biomass of *N. stricta* (Fig. 6b), as well as the maximum shoot height of dicotyledons in the experimental plots (Fig. 6c). The effect of watering the blocks, however important, only slightly influenced the *N. stricta* parameters. The mean maximum height of all plants was also significantly stimulated by low amounts of PGA, especially PGA_1_. PGA_2_ treatment decreased the three parameters to levels similar to those observed in the PGA_0_ plots. In this case, watering had only a small effect (F-values more than 10-times lower for watering than for PGA treatment (Supplement 2) (Fig. 6c)).

The regeneration of shoots of *N. stricta* expressed by DFR depended mostly on the PGA treatment (Fig. 6d). The watering had no effect on shoot regeneration only with the PGA_0.3_ concentration, where the lowest DFR values indicated the best regeneration of shoots. In the case of PGA_0_ treatment, better shoot regeneration was observed in the WW blocks, but in general the watering, even at high amounts, only slightly influenced *N. stricta* regeneration. The watering appeared much more important for dicotyledon density expressed as DMR (Fig. 6e). Both parameters, watering and PGA application at a low or medium concentration highly enhanced the appearance of dicotyledons, whereas in water stress conditions (RW blocks) no beneficial effect on the dicotyledon plant density was observed.

**Figure 7:**
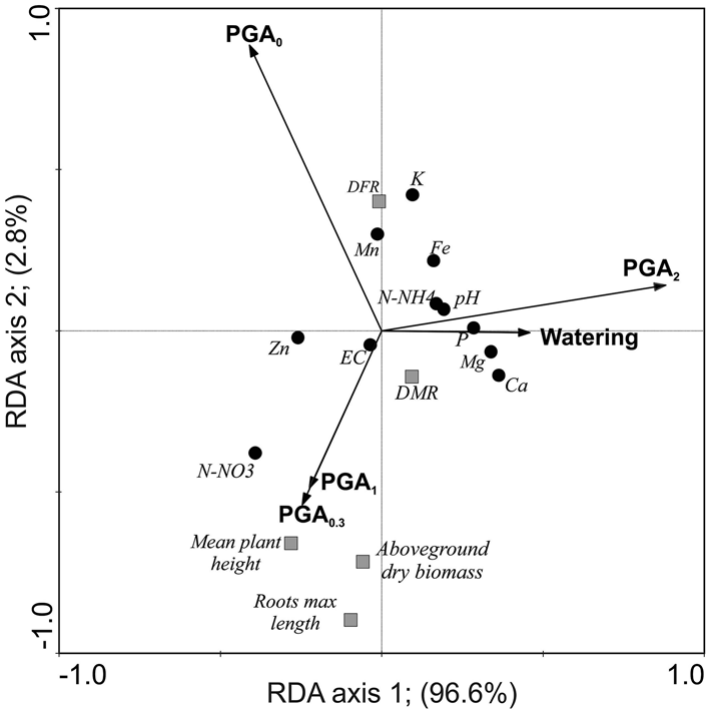
The ordination diagram for the first two axes for redundancy analysis of treatments/experimental variables (arrows), soil properties (dots), and plant life traits (squares).

Redundancy analysis revealed 96.6% of the variance of the experimental variables (watering, PGA concentration) for the first ordination axis (Fig. 7). The main gradient along the first axis was related to the PGA_2_ treatment (r = 0.26) and watering (r = 0.13), while the second gradient, describing only 2.8% of the variance, was related to PGA_0.3_ (r = − 0.20) and PGA_1_ (r = − 0.18). The concentration of PGA_2_ correlated positively with the content of most of the ions in the soil, as well as with pH, while N-NO_3_^−^ and Zn^2+^ were related to lower concentrations of PGA (PGA_0.3_ and PGA_1_). Along the PGA_0.3_ and PGA_1_ gradient, some plant functional traits such as mean plant height, root max length, and aboveground dry biomass also increased, which indicated an improvement in plant growth during drought stress. Another plant parameter, DRF, and the K^+^ content were positively related to the PGA_0_ gradient and negatively to the PGA_0.3_ and PGA_1_ gradient. This relationship indicated that the application of low doses of PGA (0.3–1%) stimulated regeneration of *N. stricta* shoots and enhanced K^+^ uptake from the soil.

## Discussion

Our studies have shown for the first time that drought stress effects in grasslands were positively mitigated by the application of specific doses of γ-PGA into light soil. We found a positive significant impact of PGA, supporting both nutrient management and plant functional traits in grasslands affected by drought. In general, the results of the research indicated that for reducing the effects of drought on meadow vegetation, the use of a certain concentration of PGA was more important than irrigation. We therefore demonstrated the biostimulatory effect of PGA in combating drought stress in grassland vegetation. Our results confirmed previous hydroponic studies for *Brassica napus*^20^ and a pot trial of *Brassica rapa* cultivars^13^.

Drought-induced stress disrupts the uptake and translocation of certain nutrients in the whole plant^25^. The more severe the drought, the more limited the flow of water and nutrients becomes, and the more limited is the availability of nutrients that can be absorbed by the roots^32^. The use of PGA can reduce this stress, most probably because it behaves as a chelator and biostimulator^13,33^ and facilitates the penetration of water and nutrients through the membranes of plant cells and the supply of nutrients in conditions of water deficit^34–36^.

The uptake of most nutrients by plants depends on the water content in the soil^37^. The nitrogen supply of plants is closely related to the availability of water, and in drought conditions nitrogen mineralization and mobility are very limited^38^. The major source of plant nitrogen is mineral nitrogen, N_min_, which occurs in two main forms (N_min_ = N-NH_4_^+^ + N-NO_3_^−^). Our results indicate that after the application of PGA, the concentration of N_min_ in the soil generally increased (Fig. 3). In the case of PGA_2_ and PGA_1_, the N_min_ content was at a medium level, while in the case of PGA_0.3_ it increased to a very high level (according to the proposed reference values for very light and light soils in Poland)^39^. N-NH_4_^+^ cations are retained in the soil by cation exchange, unlike N-NO_3_^−^, which can be leached^40,41^. In the case of sandy soils (as in our experiment) characterized by a low cation exchange capacity, the addition of PGA played an essential role in the binding of N-NH_4_^+^. At the highest concentration of PGA_2_, the N-NH_4_^+^ content was the highest. The binding of ions, including N-NH_4_^+^, is possibly due to the specific structure of this anionic biopolymer, which has numerous carboxyl groups in the side chains^9^. Some part of the NH_4_^+^ probably resulted from PGA degradation, but we assume that it was a negligible amount. Otherwise, the NH_4_^+^ content should be strongly dependent on the PGA concentration used and higher than in PGA_0_, but such an effect was not observed (Fig. 3b). The role of PGA in the “N-NH_4_^+^ turnover pool” was described by Liu et al.^42^, highlighting the regulatory role of PGA, which enables the release of ammonium ions during its deficit in plants as well as the binding of any excess. Consistent with our findings, the content of mineral forms of nitrogen, as well as the total nitrogen in the sandy clay loam soil, increased with an increase in the applied PGA dose. Such a trend was also noted in a simplified system by Zhang et al.^13^.

In soils, N-NH_4_^+^ is transformed into N-NO_3_^−^ as a result of the nitrification process^41^. Our results following PGA_2_ treatment demonstrated that the nitrification process was twice as slow relative to PGA_0_, as evidenced by the NH_4_^+^:NO_3_^−^ ratio. The mean ratio for the reference treatment was 1.30, while for PGA_2_ it was 2.95. The nitrogen nitrification process was most intense at the lowest PGA concentration. The NH_4_^+^:NO_3_^−^ ratios were significantly lower at 0.98 and 0.65 for PGA_1_ and PGA_0.3_, respectively. Similar results related to the retention and transitions over time of N-NH_4_^+^ and N-NO_3_^−^ were reported by Zhang et al.^43^. This trend suggests that when higher doses of PGA are applied on light soils, it may be possible to retain more N-NH_4_^+^ ions and reduce N-NO_3_^−^ losses due to leaching, which is consistent with the results of the studies by Zhang et al.^13^.

Due to its chelating properties, PGA forms fully water-soluble salts with K^+^, Na^+^, Ca^2+^_,_ and Mg^2+^ ions^44^. The present study showed an increase in the retention of alkaline cations by PGA, which is a positive effect, especially in light soils with a low cation exchange capacity. The content of Ca^2+^ and Mg^2+^ ions was higher if higher concentrations of PGA were used. In conditions of water deficit, Mg^2+^ is practically physiologically inaccessible to plants^45^, despite it being essential for the development of the root system and adequate root feeding^46^. Xu et al.^47^ showed that the positive effect of PGA on nitrogen metabolism and thus plant growth is related to Ca^2+^ ions. In general, under drought stress, the availability of Ca^2+^ to plants decreases, but only slightly compared to available P and K^+^^45^.

The availability of P for plants and its accumulation in biomass decreases even under moderate drought conditions^38^. In the current study, a similar tendency of available P accumulation in the soil was observed when using different PGA concentrations, as in the case of Ca^2+^ and Mg^2+^. In turn, Zhang et al.^13^ and Xue and Zhang^48^ found a decrease in the available P content in the soil after the application of PGA, which was associated with microbial immobilization. In the case of sandy soils (as in our experiment), microbial biomass (C and N) is lower compared to silty or clayey soils^49^, hence a higher content of available P after using PGA compared to PGA_0_, especially when using lower concentrations.

The K^+^ content in soil, however, was different. The highest K^+^ content was found in PGA_0_, while in the case of PGA application, its content in the soil decreased with decreasing PGA concentration, which proves its most intense uptake at lower PGA concentrations. K plays a very important role in the context of plant resistance to the effects of drought stress, as it is responsible for regulating osmotic pressure and maintaining the turgor of plant cells^50^. Its mobility, and thus bioavailability, decreases as the water content of the soil decreases^45^. Zhang et al.^43^ did not observe any effect of the application of the PGA additive on the available potassium content under watering conditions. In turn, the obtained results suggest that under stress conditions, the addition of PGA (probably the higher the concentration of PGA, the more potassium ions were bound) increased the potassium uptake by plants. This proves the importance of PGA in reducing the effects of drought stress in plants.

The use of PGA also increased the uptake of Mn^2+^ and Zn^2+^ (apart from the PGA_1_ concentration), as evidenced by lower concentrations of these ions compared to PGA_0_. Zn uptake is well illustrated by the RDA diagram, where its concentration is related to good N-NO_3_^−^ supply^51^. Also, the availability of Fe and its uptake by plants could be more closely related to the intensity of N-NO_3_^−^ and H^+^ ion uptake, which leads to an increase in the reaction in the root zone and a reduction in iron availability^52^.

The increase in the pH value in relation to PGA_0_ at PGA_2_ (also PGA_1_) can be associated with: (1) increased content of calcium and magnesium ions, or (2) increased uptake of N-NO_3_^−^, which leads to an increase in the reaction in the rhizosphere because H^+^ is transported along with NO_3_^−^. An increase in pH values by 0.1 to 0.2 units was also observed by Zhang et al.^43^. On the other hand, at PGA_0.3_, the pH dropped slightly below the value for PGA_0_, which may have been the result of the most intensive oxidation of N-NH_4_^+^ to N-NO_3_^−^ taking place at this concentration, during which hydrogen ions are released, which promotes acidification^41^.

The water content and its availability in the substrate determine the rate of nutrient supply to plants during their life cycle. Drought stress is a key factor limiting root size growth, which in turn affects nutrient uptake^38^. Since the roots are the main system that allows water to be taken from the soil, the rate of their growth, their degree of compaction, and the size they reach are of fundamental importance for plant tolerance of drought stress^53^. The architecture of the root system is closely related to the distribution of moisture and nutrients in the soil profile. The development of long roots is related to the plant's adaptation to drought conditions by its uptake of water available deeper in the profile^54^ and nutrients, such as nitrogen, that are leached to deeper layers^55^. The size of the root system is the main factor limiting the uptake of P and Ca in particular, and to a lesser extent NO_3_^−^ and K^+^^32^.

The application of PGA has a positive effect on the length and activity of plant roots^43,56^. The results of our experiments indicate that PGA stimulates the growth of the rhizosphere. The lowest concentration of PGA (PGA_0.3_) stimulated the growth of roots, as well as being responsible for a significant concentration decrease for potassium in the soil. Potassium is essential for root cell proliferation in drought conditions, which accelerates root elongation^50^. Due to the better development of the root system, the plant’s supply of nutrients and water also improved. This resulted in an increase in the yield of dry aboveground biomass and a smaller amount of dried plants compared to fresh plants, the best results being obtained with the use of the lowest concentration of PGA (PGA_0.3_).

In general, the better development of vegetation with the use of PGA_0.3_ was associated with the lowest levels of the available forms of most macro- and micronutrients in the soil (except N-NO_3_^−^) (Fig. 7), which may be due to the more efficient uptake of nutrients at the lowest PGA concentration used. At higher PGA concentrations, the effect of viscosity on the ion exchange between salt particles and soil solution, and thus on the availability of nutrients, may be important.

It was surprising that watering had no effect on soil parameters under conditions of drought stress and only a slight effect on plant parameters. Sandy soils exhibit a high filtration index^57^, and even when watering was carried out with different PGA concentrations, no positive effect was noted. The only exception was the increase in the share of dicotyledonous plants in the plots studied, their survival and germination on PGA plots being further enhanced by watering. Drought stress is also responsible for the reduction in growth of perennials in agricultural and seminatural habitats^58–60^. Drought immobilizes the seeds in the soil as a survival adaptation to drought stress^61^. As a consequence, prolonged drought events influence negatively on plant diversity, ecosystem functioning, and the available resources for pollinators^25,26^. In our studies, a low concentration of PGA increased the DMR. This parameter was also accelerated by the watering conditions. These results indicate that PGA accelerates the dicotyledons’ growth, but it is necessary to water during the growing season, and even giving 30% of the calculated water deficit for a given month in a multi-year period increased the DMR.

PGA is a biopolymer that is produced by bacteria as a biofilm component in artificial conditions, but should be treated as a natural element of ecosystems^25,62^. Its presence in agrosystems is reduced due to the elimination of many bacterial strains during agricultural activities and soil changes^63^. Artificial supplementation of PGA into agroecosystems is beneficial for crops^13,51^, but it has not been previously applied to grasslands. We showed that precise amounts of PGA enhance plant growth and the regeneration of shoots during drought periods. We do not believe that it directly holds water as a hydrogel, but it can be regarded as an effective biostimulator^64^, helping the plant to grow and survive through dry periods. These data are preliminary and studies should be continued using different soil conditions and also on intensively managed grasslands, as well as those of conservation value.

## Conclusions

The results of our field experiment combining watering and the application of different concentrations of γ-PGA to grassland during a period of drought clearly showed that PGA acts as biostimulant, alleviating the effects of stress in plants. The biostimulation effect of PGA consisted in increasing the availability of ions, especially K^+^, Zn^2+^, Mn^2+^, Fe^2+/3+^, Ca^2+^, Mg^2+^, and mineral forms of nitrogen (N-NH_4_^+^ and N-NO_3_^−^), elongating plant roots, and increasing the aboveground biomass, as well as improving the resprouting capacity of the dominant grass, *N. stricta*, and the regeneration of dicotyledons. In the case of meadows on sandy soils, the use of low PGA concentrations (0.3% or 1%) was the most beneficial for both the availability of macro- and microelements and for plant functional traits. Irrigation had a greater effect than using PGA only for the dicotyledon to monocotyledon ratio (DMR). Again, it was most advantageous to use the lowest concentration of PGA, but in combination with watering. On the basis of the obtained results, we recommend using a PGA concentration in the range of 0.3–1% to manage grassland on light soils. PGA in the given doses is a promising treatment, which protects grassland ecosystems from drought stress.

### Supplementary Information

Supplementary Information 1.

Supplementary Information 2.

## Data Availability

The data supporting the findings of this study are available from the corresponding author upon reasonable request.
