# Supplementary material for: Effects of γ-polyglutamic acid on grassland sandy soil properties and plant functional traits exposed to drought stress
Source: Sci Rep. 2024 Feb 14;14:3769. doi: 10.1038/s41598-024-54459-1 (PMC10866894; doi:10.1038/s41598-024-54459-1)
Supplement: Supplementary file 1 — Supplementary Information 1. [file 41598_2024_54459_MOESM1_ESM.docx]

**Supplement 1**

**Two-way analysis of variance of soil parameters in experimental design: watering blocks and treatment (PGA content)**

| Effect | SS | d.f | MS | F | p |
| --- | --- | --- | --- | --- | --- |
| Ca |  |  |  |  |  |
| **Intercept** | **6451592** | **1** | **6451592.00** | **1932.54** | **0.0000** |
| **Treatment** | **45549** | **3** | **15183.00** | **4.55** | **0.0053** |
| Watering Blocks | 2098 | 2 | 1049.00 | 0.31 | 0.7312 |
| Treatment* Watering Blocks | 18965 | 6 | 3161.00 | 0.95 | 0.4663 |
| Error | 280426 | 84 | 3338.00 |  |  |
| Mg |  |  |  |  |  |
| **Intercept** | **44699.1** | **1** | **44699.09** | **310.45** | **0.0000** |
| **Treatment** | **1811.58** | **3** | **603.86** | **4.19** | **0.0081** |
| Watering Blocks | 133.03 | 2 | 66.52 | 0.46 | 0.6316 |
| Treatment* Watering Blocks | 1463.89 | 6 | 243.98 | 1.69 | 0.1323 |
| Error | 12094.3 | 84 | 143.98 |  |  |
| K |  |  |  |  |  |
| **Intercept** | **89823.3** | **1** | **89823.25** | **1633.58** | **0.0000** |
| **Treatment** | **1820.79** | **3** | **606.93** | **11.04** | **0.0000** |
| Watering Blocks | 64.81 | 2 | 32.40 | 0.59 | 0.5570 |
| Treatment* Watering Blocks | 398.12 | 6 | 66.35 | 1.21 | 0.3110 |
| Error | 4618.78 | 84 | 54.99 |  |  |
| P |  |  |  |  |  |
| **Intercept** | **606442** | **1** | **606441.90** | **329.98** | **0.0000** |
| **Treatment** | **147243** | **3** | **49080.90** | **26.71** | **0.0000** |
| Watering Blocks | 11068.9 | 2 | 5534.40 | 3.01 | 0.0546 |
| Treatment* Watering Blocks | 21523.4 | 6 | 3587.20 | 1.95 | 0.0818 |
| Error | 154378 | 84 | 1837.80 |  |  |
| N-NH4 |  |  |  |  |  |
| **Intercept** | **7236.69** | **1** | **7236.69** | **461.62** | **0.0000** |
| **Treatment** | **63.023** | **3** | **21.01** | **1.34** | **0.2669** |
| Watering Blocks | 14.716 | 2 | 7.36 | 0.47 | 0.6270 |
| Treatment* Watering Blocks | 90.884 | 6 | 15.15 | 0.97 | 0.4532 |
| Error | 1316.84 | 84 | 15.68 |  |  |
| N-NO3 |  |  |  |  |  |
| **Intercept** | **5762.9** | **1** | **5762.90** | **371.83** | **0.0000** |
| **Treatment** | **1178.12** | **3** | **392.71** | **25.34** | **0.0000** |
| Watering Blocks | 2.696 | 2 | 1.35 | 0.09 | 0.9168 |
| Treatment* Watering Blocks | 45.966 | 6 | 7.66 | 0.49 | 0.8109 |
| Error | 1301.88 | 84 | 15.50 |  |  |
| Zn |  |  |  |  |  |
| **Intercept** | **204.167** | **1** | **204.17** | **1194.08** | **0.0000** |
| **Treatment** | **6.5258** | **3** | **2.18** | **12.72** | **0.0000** |
| Watering Blocks | 0.1602 | 2 | 0.08 | 0.47 | 0.6276 |
| Treatment* Watering Blocks | 0.3248 | 6 | 0.05 | 0.32 | 0.9267 |
| Error | 14.3625 | 84 | 0.17 |  |  |
| Mn |  |  |  |  |  |
| **Intercept** | **231.26** | **1** | **231.26** | **303.74** | **0.0000** |
| **Treatment** | **12.7137** | **3** | **4.24** | **5.57** | **0.0016** |
| Watering Blocks | 2.7315 | 2 | 1.37 | 1.79 | 0.1726 |
| Treatment* Watering Blocks | 2.5594 | 6 | 0.43 | 0.56 | 0.7607 |
| Error | 63.955 | 84 | 0.76 |  |  |
| pH |  |  |  |  |  |
| **Intercept** | **3162.05** | **1** | **3162.05** | **109917.30** | **0.0000** |
| **Treatment** | **0.533** | **3** | **0.18** | **6.20** | **0.0008** |
| Watering Blocks | 0.112 | 2 | 0.06 | 1.90 | 0.1491 |
| Treatment* Watering Blocks | 0.082 | 6 | 0.01 | 0.50 | 0.8246 |
| Error | 2.416 | 84 | 0.03 |  |  |
| EC |  |  |  |  |  |
| **Intercept** | **362707** | **1** | **362707.40** | **1183.44** | **0.0000** |
| Treatment | 1604 | 3 | 534.70 | 1.75 | 0.1641 |
| Watering Blocks | 144.2 | 2 | 72.10 | 0.24 | 0.7908 |
| Treatment* Watering Blocks | 2551.8 | 6 | 425.30 | 1.39 | 0.2291 |
| Error | 25744.8 | 84 | 306.50 |  |  |
