# Supplementary material for: Effects of γ-polyglutamic acid on grassland sandy soil properties and plant functional traits exposed to drought stress
Source: Sci Rep. 2024 Feb 14;14:3769. doi: 10.1038/s41598-024-54459-1 (PMC10866894; doi:10.1038/s41598-024-54459-1)
Supplement: Supplementary file 2 — Supplementary Information 2. [file 41598_2024_54459_MOESM2_ESM.docx]

**Supplement 2**

**Two way analysis of variance for Blocks (Watering) and Treatments (PGA amounts)**

| Effect | SS | d.f | MS | F | p |
| --- | --- | --- | --- | --- | --- |
| **Root max length** |  |  |  |  |  |
| Intercept | 149932.7 | 1 | 149932.70 | 36037.32 | 0.00000 |
| Treatment | 16137.4 | 3 | 5379.10 | 1292.91 | 0.00000 |
| Watering Blocks | 105.4 | 2 | 52.70 | 12.67 | 0.00000 |
| Treatment* Watering Blocks | 124 | 6 | 20.70 | 4.97 | 0.00005 |
| Error | 3145.3 | 756 | 4.20 |  |  |
| **Mean dry biomass** |  |  |  |  |  |
| Intercept | 7752.083 | 1 | 7752.08 | 4144.17 | 0.00000 |
| Treatment | 1898.803 | 3 | 632.93 | 338.36 | 0.00000 |
| Watering Blocks | 36.568 | 2 | 18.28 | 9.77 | 0.00006 |
| Treatment* Watering Blocks | 101.718 | 6 | 16.95 | 9.06 | 0.00000 |
| Error | 1414.175 | 756 | 1.87 |  |  |
| **Dry:Fresh ratio** |  |  |  |  |  |
| Intercept | 5991.41 | 1 | 5991.41 | 286.36 | 0.00000 |
| Treatment | 3387.09 | 3 | 1129.03 | 53.96 | 0.00000 |
| Watering Blocks | 414.84 | 2 | 207.42 | 9.91 | 0.00006 |
| Treatment* Watering Blocks | 746.01 | 6 | 124.34 | 5.94 | 0.00000 |
| Error | 15817.73 | 756 | 20.92 |  |  |
| **Mean max Height** |  |  |  |  |  |
| Intercept | 363877.7 | 1 | 363877.70 | 8512.71 | 0.00000 |
| Treatment | 41891 | 3 | 13963.70 | 326.67 | 0.00000 |
| Watering Blocks | 189.1 | 2 | 94.50 | 2.21 | 0.11023 |
| Treatment* Watering Blocks | 733.8 | 6 | 122.30 | 2.86 | 0.00921 |
| Error | 32315.4 | 756 | 42.70 |  |  |
| **DICO/MONOCOT ratio** |  |  |  |  |  |
| Intercept | 4754.099 | 1 | 4754.10 | 392.07 | 0.00000 |
| Treatment | 256.041 | 3 | 85.35 | 7.04 | 0.00011 |
| Watering Blocks | 872.881 | 2 | 436.44 | 35.99 | 0.00000 |
| Treatment* Watering Blocks | 335.997 | 6 | 56.00 | 4.62 | 0.00013 |
| Error | 9166.95 | 756 | 12.13 |  |  |
